# The use of high resolution anterior segment optical coherence tomography for the characterization of conjunctival lymphoma, conjunctival amyloidosis and benign reactive lymphoid hyperplasia

**DOI:** 10.1186/s40662-019-0143-4

**Published:** 2019-06-18

**Authors:** Nandini Venkateswaran, Carolina Mercado, Ann Q. Tran, Armando Garcia, Pedro Francisco Monsalve Diaz, Sander R. Dubovy, Anat Galor, Carol L. Karp

**Affiliations:** 1 Bascom Palmer Eye Institute, University of Miami Miller School of Medicine, 900 NW 17th Street, Miami, FL 33136 USA; 2 Department of Ophthalmology, Miami Veterans Administration Medical Center, Miami, FL USA

**Keywords:** High resolution anterior segment optical coherence tomography, Ocular surface imaging, Ocular surface lesions, Conjunctival lymphoma, Conjunctival amyloidosis, Benign reactive lymphoid hyperplasia

## Abstract

**Background:**

Conjunctival lymphoma, conjunctival amyloidosis and benign reactive lymphoid hyperplasia (BRLH) are conditions that often have a similar appearance on the ocular surface. The use of high resolution anterior segment optical coherence tomography (HR-OCT) enables clinicians to evaluate distinctive differences in tissue morphology and cellular patterns in various ocular surface conditions. In this study, we characterize the morphological differences seen in conjunctival lymphoma, conjunctival amyloidosis and BRLH on HR-OCT imaging.

**Methods:**

A retrospective chart review was performed of patients with biopsy proven conjunctival lymphoma, conjunctival amyloidosis and BRLH between 2012 and 2019 at the Bascom Palmer Eye Institute. Patients were excluded if HR-OCT imaging was not performed on initial presentation.

**Results:**

Thirty-four total eyes of 27 patients were identified. Twenty eyes had conjunctival lymphoma (16 patients), 8 eyes had conjunctival amyloidosis (6 patients) and 6 eyes had BRLH (5 patients). All conditions appeared clinically as pink, red or yellow subepithelial lesions but had different features on HR-OCT. In lymphoma, HR-OCT images typically showed homogenous, dark subepithelial lesions with smooth borders, containing monomorphic dot-like infiltrates. HR-OCT images of amyloidosis typically showed heterogeneous, dark lesions with irregular borders, often containing hyperreflective linear infiltrates. HR-OCT images of BRLH showed variable infiltration of the subepithelial tissue, at times with homogenous lesions containing dot-like infiltrates like lymphoma and other times with more hyperreflective, subepithelial tissue. Flow cytometry and gene rearrangement was needed for final differentiation between BRLH and lymphoma lesions.

**Conclusions:**

Distinctive features on HR-OCT of conjunctival lymphoma, conjunctival amyloidosis and BRLH can help characterize these lesions beyond what is apparent with the clinical examination. Future studies can further validate this technology’s use with more subtle and challenging lesions.

## Background

Conjunctival lymphoma, conjunctival amyloidosis and benign reactive lymphoid hyperplasia (BRLH) are all ocular surface conditions that can have a similar appearance on the ocular surface [1]. The diagnosis of and differentiation between these lesions is important as they can carry a significant risk of morbidity and mortality [2]. The gold standard for diagnosis is the histopathological and cytologic examination of incisional or excisional conjunctival biopsy specimens. Biopsies are however invasive diagnostic techniques that can at times be negative if inadequate tissue is obtained [3]. Now, with the advent of high resolution anterior segment optical coherence tomography (HR-OCT), an “optical biopsy” can be obtained of the eye in the office setting [4].

Images obtained by HR-OCT devices enable clinicians to evaluate for distinctive differences in tissue morphology and cellular patterns in various ocular surface conditions [5]. We have previously demonstrated the ability of HR-OCT to differentiate between epithelial malignancies (i.e. ocular surface squamous neoplasia) from subepithelial benign ocular surface lesions (i.e. pterygium) [6]. We have further demonstrated the ability to rule in or rule out malignancy even in the setting of complex ocular surface conditions [7].

While many studies have confirmed the utility of HR-OCT for epithelial malignancies, a gap exists concerning the utility of HR-OCT in differentiating between various subepithelial lesions. We have previously described HR-OCT findings in a small number of patients with conjunctival lymphoma and conjunctival amyloidosis but a systematic evaluation of the differences of HR-OCT findings has not been studied. In addition, HR-OCT findings of BRLH was not described in these previous studies [6, 8]. As such, larger studies are needed to evaluate the utility of HR-OCT in differentiating between conjunctival lymphoma, conjunctival amyloidosis and BRLH. In this study, we retrospectively reviewed cases of 34 eyes with pre-treatment HR-OCT imaging. Our goal was to determine if HR-OCT could be used as an adjunctive non-invasive diagnostic modality to guide the diagnosis and management of these subepithelial disorders.

## Methods

The institutional review board of the University of Miami approved this retrospective study, and the methods adhered to the tenets of the Declaration of Helsinki and were compliant with the Health Insurance Portability and Accountability Act.

A clinical database was used to identify patients with biopsy proven conjunctival lymphoma, conjunctival amyloidosis, and benign reactive lymphoid hyperplasia by histopathology and/or cytology at the Bascom Palmer Eye Institute between the years of January 2012 to February 2019. Retrospective chart reviews were conducted. Patients were excluded if they had no baseline HR-OCT imaging. Baseline pre-treatment and serial HR-OCT images were reviewed for all included patients. Imaging characteristics were noted and summarized.

OCT imaging was performed with two spectral domain OCT machines, the Optovue Avanti (Fremont, CA) and the Optovue RT Vue (Fremont, CA). The Optovue Avanti has a transverse resolution of 15 μm, an axial resolution of 5 μm, wavelength of 840 nm, and a scanning speed of 70,000 A-scans per second. The Optovue RT Vue has a transverse resolution of 8 μm, an axial resolution of 5 μm, wavelength of 840 nm and a scanning speed of 26,000 A-scans per second. Multiple scans were taken of each lesion, and images were reviewed by 1 of the authors (CLK). OCT scans were evaluated in terms of thickness, morphology and reflectivity of the epithelial and subepithelial layers. Hyperreflectivity was defined as increased whiteness compared to tissue of the same location as seen in normal subjects. Hyporeflectivity was defined as increased darkness compared to tissue of the same location seen in normal subjects. Thickness measurements of the epithelial and subepithelial layers were obtained using the internal caliper measurement tool in the OCT imaging analysis software.

Conjunctival biopsy specimens were sent for histopathological and cytological analysis in all cases. Both formalin fixed and fresh tissue samples were obtained in all cases. One part of the biopsy specimen was fixed in 10% buffered formalin, dehydrated and embedded in paraffin blocks. The blocks were sectioned at 5 μm and stained with hematoxylin-eosin, periodic acid Schiff, Congo-Red and other indicated stains. These stains were analyzed using a light microscope (Olympus Optical Co., Tokyo, Japan) and were photographed using a digital system. Fresh tissue specimens were also sent for cytological analysis to assess for monoclonal or polyclonal lymphocyte proliferation as well as for gene re-arrangement studies.

Descriptive statistics were used to summarize demographic information, tumor characteristics, and HR-OCT findings. One-way ANOVA analyses were used to compare the thickness of the epithelial and subepithelial layers for each condition.

## Results

### Demographic information

Thirty-four eyes of 27 patients were identified for the study. Overall, the mean age of the patient population was 61.9 ± 21.1 years, 59.2% were female, and 74% were white. Demographic information for all patients can be found in Table 1.

**Table 1:** Patient demographics by ocular surface lesion

	Conjunctival Lymphoma (*n* = 16 patients)	Conjunctival Amyloidosis (*n* = 6 patients)	Benign Reactive Lymphoid Hyperplasia (*n* = 5 patients)
Age, mean (range) (years)	63.06 (26–89)	70.7 (63–82)	47.5 (12–85)
Gender, Male, n (%)	5 (31.2)	3 (50.0)	4 (80.0)
Race, White, n (%)	10 (62.5)	5 (100.0)	4 (80.0)
Ethnicity, Hispanic, n (%)	6 (37.5)	1 (20.0)	1 (20.0)

### Conjunctival lymphoma

Twenty eyes of 16 patients with biopsy proven conjunctival lymphoma were identified. Fourteen patients had a diagnosis of mucosa-associated lymphoid tissue (MALT) lymphoma, 1 had a diagnosis of high-grade B-cell lymphoma and 1 had a diagnosis of small lymphocytic lymphoma. Of the 4 patients who had bilateral disease, all of them were MALT lymphoma. All patients were co-managed with a specialist in hematology and oncology. Systemic involvement was found in five patients who were treated with systemic chemotherapy and 10 patients were treated with external beam radiation. Some patients additionally received oral doxycycline (*n* = 5), systemic chemotherapy (*n* = 5), or intralesional interferon alpha-2b (*n* = 1).

### Conjunctival amyloidosis

Eight eyes of 6 patients with biopsy proven conjunctival amyloidosis were identified. Two patients had bilateral disease. All patients were referred to a rheumatologist to evaluate for systemic amyloidosis which was negative in all cases.

In terms of local management, 5 patients elected observation. One patient underwent surgical excision with amniotic membrane transplant to reduce discomfort from the bulky size of the lesion. The lesion subsequently recurred 1.5 years later.

### Benign reactive lymphoid hyperplasia

Six eyes of 5 patients referred for possible conjunctival lymphoma had biopsy proven BRLH. One patient had bilateral disease. One patient had a fellow eye with MALT lymphoma. Two patients were treated with oral doxycycline, one of whom had complete resolution of the lesion. Two patients, ages 13 and 14, underwent excisional biopsy with no signs of recurrence at 1.5 years. Two patients were observed without treatment following the incisional biopsy.

### Clinical, HR-OCT, and histopathological characteristics

Clinically, all 3 conditions appeared as pink, red or yellow subepithelial lesions on examination but displayed different optical features on HR-OCT as summarized in Table 2. All patients had a normal epithelial layer on HR-OCT images. However, in the subepithelial region, more distinctive findings were noted.

**Table 2:** HR-OCT findings of conjunctival lymphoma, conjunctival amyloidosis and benign reactive lymphoid hyperplasia

Lesion	Epithelial Layer	Subepithelial Layer
Conjunctival Lymphoma	Normal	Subepithelial lesion that is hyporeflective, homogenous, with smooth borders containing monomorphic dot-like infiltrates bordered superiorly and inferiorly by a hyperreflective band underneath the epithelium of variable thickness
Conjunctival Amyloidosis	Normal	Subepithelial lesion that is heterogeneous with irregular borders containing hyperreflective linear opacities
Benign Reactive Lymphoid Hyperplasia	Normal	Variably reflective subepithelial tissue No hyperreflective band seen underneath the epithelium in paucicellular cases but present in hypercellular cases

### Conjunctival lymphoma

**Fig. 1:**
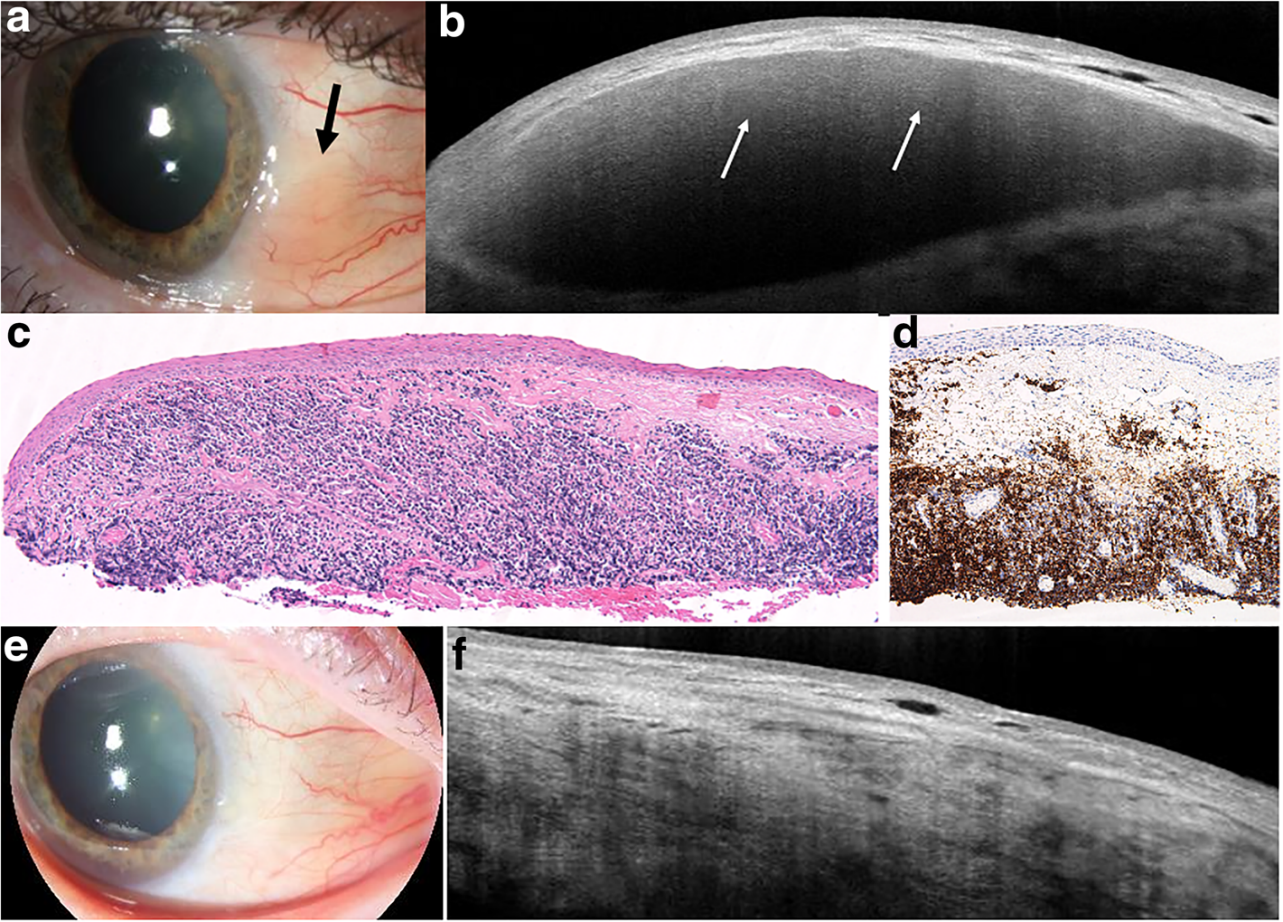
Slit lamp and HR-OCT images and histopathology of conjunctival lymphoma. **a** Slit lamp photograph of a 72-year-old male who presented with a pink, subepithelial nodule on the bulbar conjunctiva in the left eye (black arrow). **b** HR-OCT through the lesion revealed a normal epithelium but the presence of a large, hyporeflective, homogenous appearing subepithelial lesion infiltrated with monomorphic dot-like infiltrates (white arrows). A hyperreflective band of tissue is noted above and below the infiltrate. **c** HR-OCT images corresponded with histopathology, in which a moderately dense, monomorphic lymphocytic cell population was present within the substantia propria (hematoxylin and eosin; original magnification × 100). **d** Immunohistochemical studies were positive for CD20 (shown in image; original magnification × 100) and negative for C5 and CD10. The plasma cells were monoclonal for kappa light chain by in situ hybridization (ISH), consistent with mucosa-associated lymphoid tissue (MALT) lymphoma. **e** After 17 sessions of external beam radiation therapy, the subepithelial lesion resolved clinically. **f** On HR-OCT, there was complete normalization of the conjunctival architecture with resolution of the subepithelial lesion

**Fig. 2:**
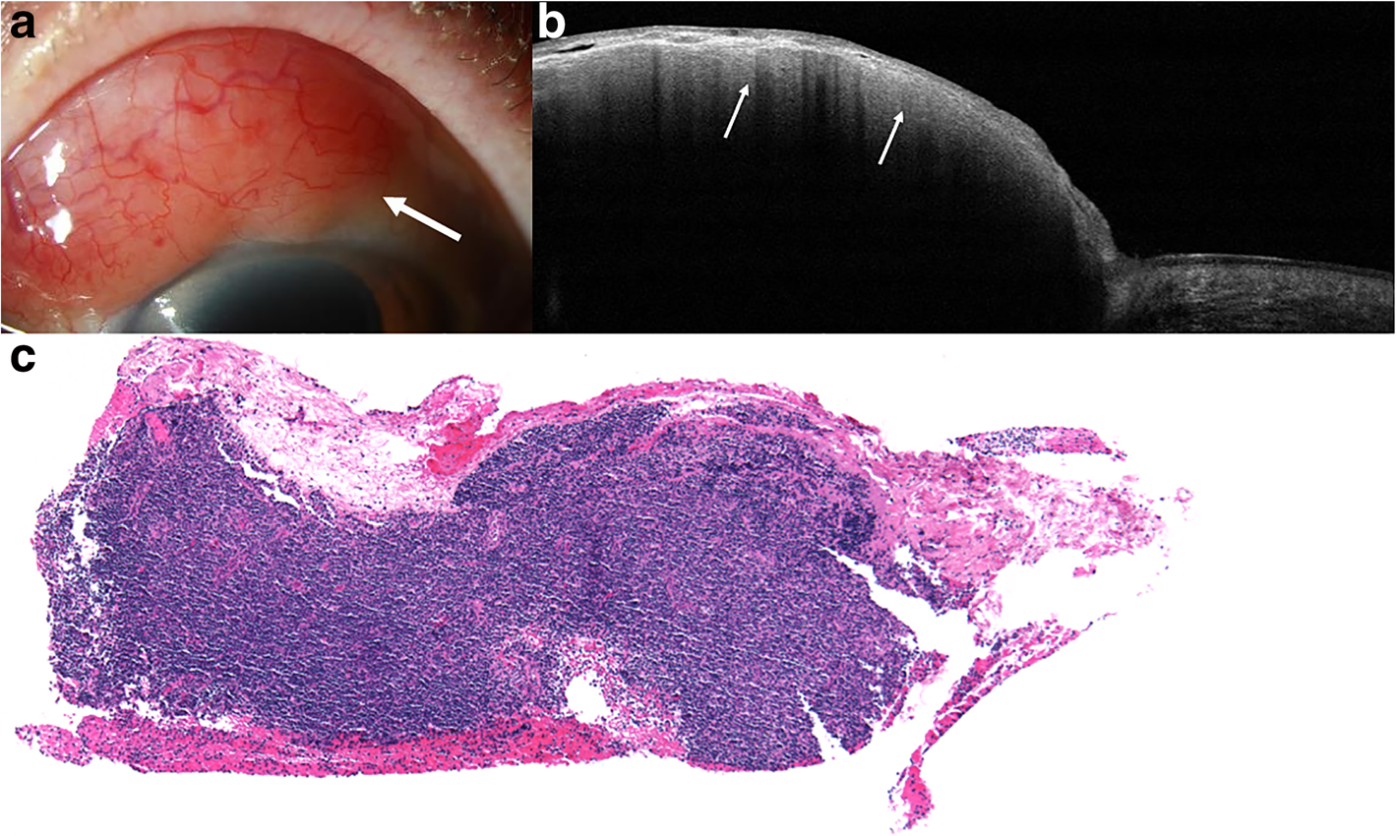
Slit lamp and HR-OCT images and histopathology of conjunctival lymphoma. **a** Slit lamp photograph of an 86-year-old female who presented with a red-pink salmon patch lesion superiorly in the bulbar conjunctiva of the left eye (white arrow). **b** HR-OCT through the lesion revealed a normal epithelial layer with a subepithelial, hyporeflective, homogenous lesion with monomorphic dot-like infiltrates (white arrows). Note the hyperreflective band beneath the epithelium. **c** A dense subepithelial lymphocytic infiltrate was present within the substantia propria (hematoxylin and eosin; original magnification 100x). Immunohistochemistry revealed a monoclonal B-cell proliferation that consisted of a CD20 and CD19 positive B-cell proliferation positive for BCL2, co-expression of CD5 and lambda surface light chain restriction by ISH, all consistent with small lymphocytic lymphoma

In lymphoma, HR-OCT images typically showed homogenous, dark subepithelial lesions with smooth borders, with monomorphic dot-like infiltrates as seen in Figs. 1 and 2. The subepithelial lesions were typically bordered by a hyperreflective band underneath the epithelium. HR-OCT in 19 of the 20 eyes with conjunctival lymphoma had these distinctive features. One eye showed findings more similar to those of BRLH, with thickened, hyperreflective subepithelial tissue as opposed to a discrete, homogenous, hyporeflective, subepithelial lesion. All were confirmed to be lymphoma by histopathology. The hyperreflectivity in one case was likely because this patient presented with recurrent subconjunctival hemorrhages and the lesion on HR-OCT was surrounded by hemorrhage.

Histopathology and flow cytometry confirmed monoclonal cell infiltration in all cases corresponding to the monomorphic dot-like infiltrates seen on HR-OCT. Eighteen eyes were MALT lymphomas. One case was confirmed to be diffuse large B-cell lymphoma and another small lymphocytic leukemia by flow cytometry. The mean epithelial thickness of all lesions was 65.3 ± 31.6 μm and mean subepithelial lesion thickness was 632.6 ± 311.7 μm by in vivo OCT imaging.

### Conjunctival amyloidosis

**Fig. 3:**
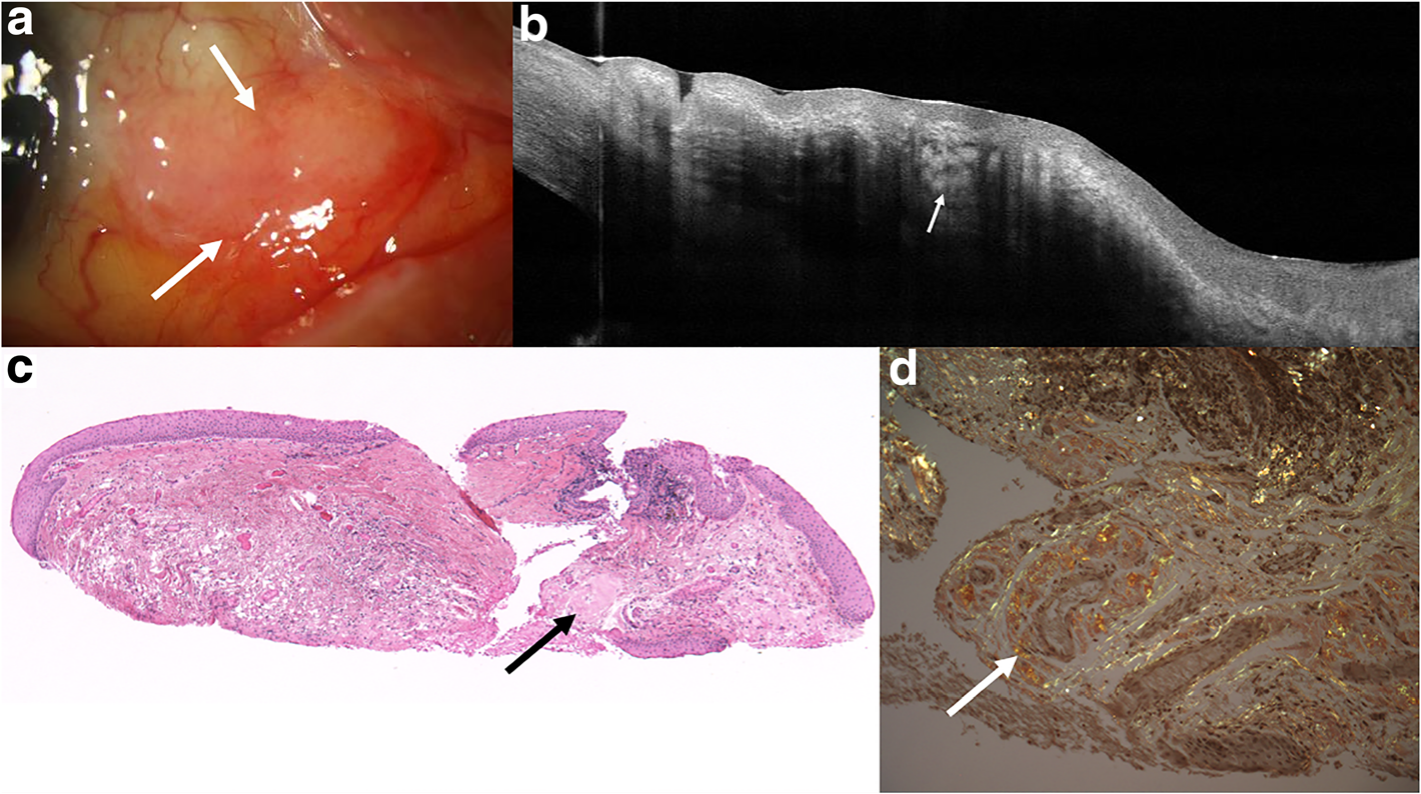
Slit lamp and HR-OCT images and histopathology of conjunctival amyloidosis. **a** Slit lamp photograph of a 71-year-old female who presented with bilateral bulbar conjunctival lesions that were yellow-red and fleshy in appearance, more prominent in the right eye (right eye lesion featured, white arrows). **b** HR-OCT through the lesion in the right eye showed a normal epithelial layer with a large heterogeneous, subepithelial lesion containing hyperreflective opacities consistent with deposition of amyloid material (white arrow). **c** Paucicellular amorphous material consistent with amyloid is present within the substantia propria (black arrow) that corresponds with the subepithelial hyperreflective opacities on OCT (hematoxylin and eosin; original magnification × 100). **d** The amyloid material (white arrow) demonstrates apple-green birefringence (Congo red; original magnification × 200)

**Fig. 4:**
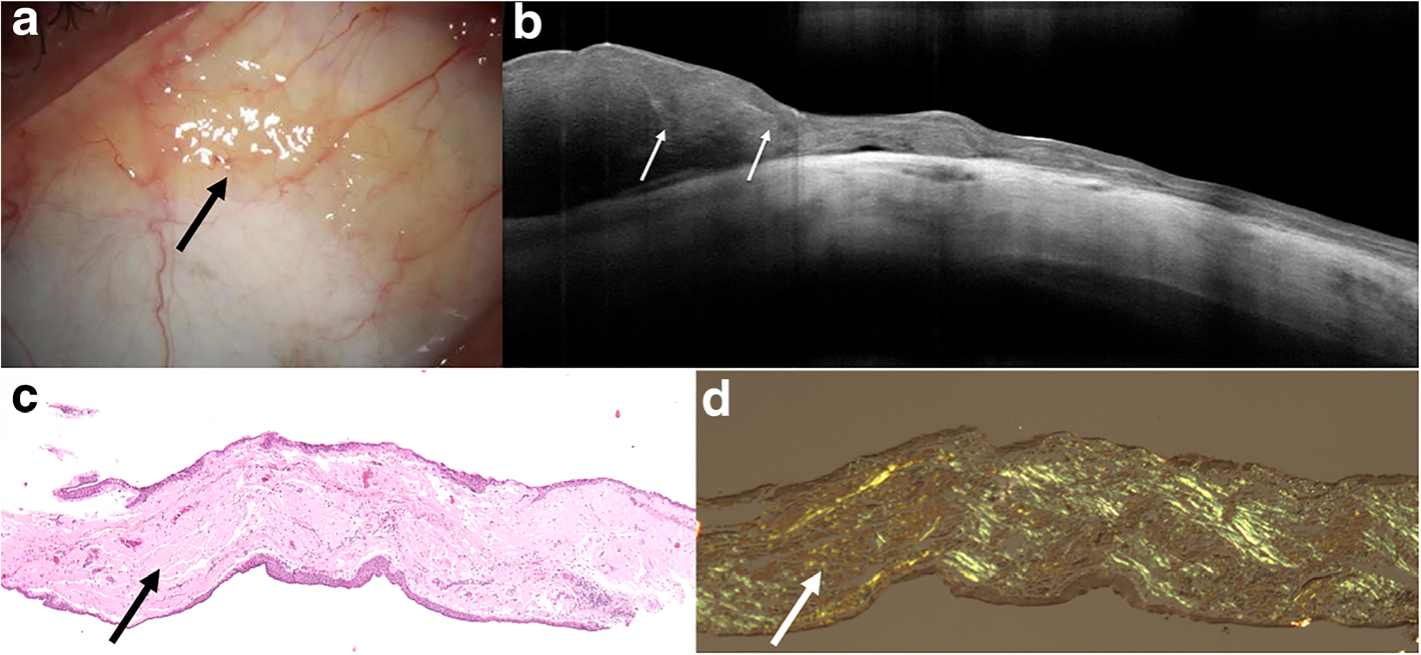
Slit lamp and HR-OCT images and histopathology of conjunctival amyloidosis. **a** Slit lamp photograph of a 66-year-old male who presented with a gelatinous lesion on the bulbar conjunctiva on the left eye (black arrow). **b** HR-OCT through the lesion revealed a normal epithelial layer with a heterogeneous, subepithelial lesion with several hyperreflective opacities (white arrows). **c** Paucicelluar amorphous material consistent with amyloid was present within the substantia propria (black arrow) (hematoxylin and eosin; original magnification × 100). **d** The amyloid material demonstrates apple-green birefringence (white arrow) (Congo red; original magnification × 100)

In contrast, HR-OCT images of amyloidosis typically showed heterogeneous, dark lesions with irregular borders, often containing hyperreflective linear infiltrates as seen in Figs. 3 and 4. HR-OCT images of all 8 eyes with conjunctival amyloidosis had these distinctive features. Histopathology confirmed amyloid deposition in all cases. Mean epithelial thickness of all lesions was 54.3 ± 20.4 μm and mean subepithelial lesion thickness was 563.2 ± 136.9 μm by in vivo OCT imaging.

### Benign reactive lymphoid hyperplasia

**Fig. 5:**
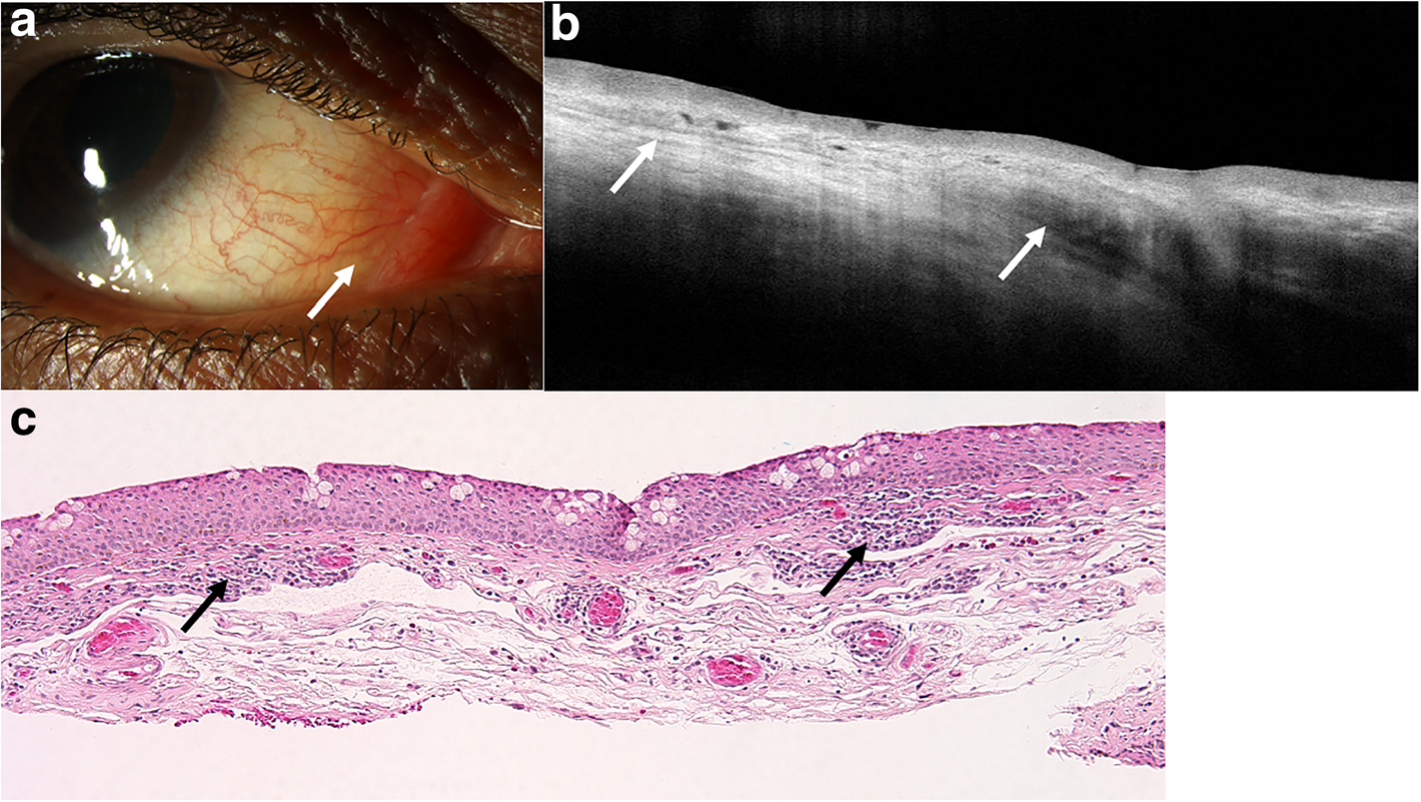
Slit lamp and HR-OCT images and histopathology of benign reactive lymphoid hyperplasia. **a** Slit lamp photograph of an 80-year-old dark skinned male who presented with an area of nasal hyperemia in the right eye near the caruncular region (white arrow). His fellow eye had a diffuse salmon patch lesion that was biopsy-proven MALT lymphoma. **b** HR-OCT revealed an OCT with minimal hyporeflective infiltrate (white arrows) within the overall hyperreflective subepithelial tissue. **c** A paucicellular infiltrate of moderate sized lymphocytes is present within the substantia propria (black arrows). No morphologic or immunohistochemical evidence of lymphoma is present (hematoxylin and eosin, original magnification × 100) and flow cytometry was negative for B or T cell proliferation, which was consistent with benign reactive lymphoid hyperplasia

**Fig. 6:**
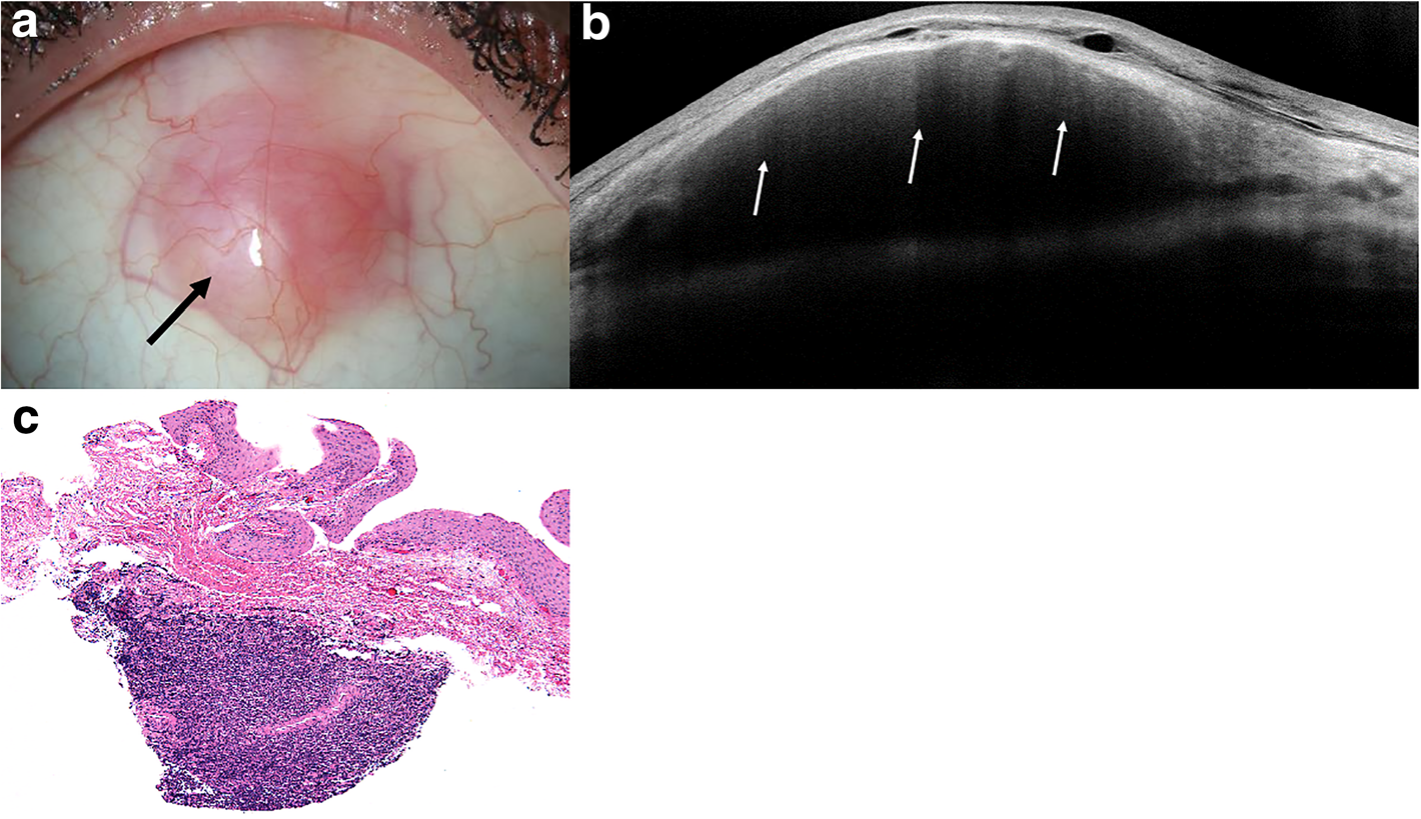
Slit lamp and HR-OCT images and histopathology of benign reactive lymphoid hyperplasia. **a** Slit lamp photograph of a 49-year-old Caucasian female who presented with a superior pink bulbar conjunctival lesion (black arrow). **b** HR-OCT revealed a normal epithelial layer with a large, homogenous, hyporeflective subepithelial lesion with monomorphic dot-like infiltrates (white arrows). Note the hyperreflective band of tissue below the epithelium and above the infiltrate. **c** A hypercellular monomorphic infiltrate was present within the substantia propria (hematoxylin and eosin; original magnification × 100). Flow cytometry was negative for B or T cell proliferation. Given its appearance clinically and on HR-OCT, this lesion was biopsied 3 separate times, all of which corroborated the diagnosis of benign reactive lymphoid hyperplasia

HR-OCT images of BRLH were similar to that of lymphoma, with lesions in the subepithelial tissue. These subepithelial lesions also had a monomorphic infiltrate but were more hyperreflective in BRLH cases compared to the lymphoma images as seen in Fig. 5. The hyperreflective subepithelial lesions on HR OCT corresponded to a paucicellular infiltrate compared to the hypercellular infiltrates typically seen with lymphoma. Two of the eyes had larger, hyporeflective subepithelial lesions with discrete borders that appeared more similar to HR-OCT images of conjunctival lymphoma as seen in Fig. 6. These cases had high levels of cellular infiltrates. Thus, the BRLH cases were more variable in character.

Histopathological analysis of BRLH lesions showed a hypercellular infiltrate in lesions that appeared similar to conjunctival lymphoma on HR-OCT and a paucicellular infiltrate in those lesions that showed more hyperreflective subepithelial tissue. Flow cytometry was negative for B or T cell proliferation in all cases. Mean epithelial thickness of all lesions was 146.8 ± 113.2 μm and mean subepithelial lesion thickness was 601.0 ± 148.8 μm by in vivo OCT imaging.

### Lymphoma, amyloidoisis, and benign reactive lymphoid hyperplasia comparison

Overall, the amyloid lesions were easily distinguishable from the lymphoma cases. The lymphoma cases were consistently hyporeflective with dark dot-like infiltrates within a hyperreflective band beneath the epithelium. The benign lymphoproliferative lesions were more variable on HR-OCT reflecting the variable cellular infiltration but were distinct from the findings seen in amyloid lesions. In addition to traditional pathology, flow cytometry and gene rearrangement was needed for final differentiation of the lymphoproliferative lesions.

One-way ANOVA analysis showed a statistical difference between epithelial layer thickness in conjunctival lymphoma and benign lymphoid hyperplasia (*p* = 0.027). No statistically significant differences were noted between groups when comparing subepithelial lesion thickness.

## Discussion

Conjunctival lymphoma, conjunctival amyloidosis and BRLH are all ocular surface conditions that can appear very similar clinically. All three entities can manifest as a focal salmon patch or waxy, yellow, red or pink appearing lesions [1, 2]. As such, differentiating among these entities can prove to be challenging, and this can delay appropriate treatment. In this study, we assessed the utility of HR-OCT in the diagnosis and differentiation of these subepithelial diseases of the conjunctiva. HR-OCT was able to successfully distinguish lymphoproliferative lesions from amyloid lesions; however, it was not always able to distinguish between benign and malignant lymphoproliferative lesions.

On HR-OCT, conjunctival lymphoma appeared as homogeneous, hyporeflective subepithelial lesions with regular borders that contained monomorphic dot-like infiltrates. The lesions were often bordered superiorly and inferiorly by a band of hyperreflectivity that either represents changes in reflectivity from the interface between the epithelial and subepithelial tissue or mechanical displacement of the hyperreflective substantia propria by the lymphocytic infiltrate. The dot-like infiltrates contained within the lesions on HR-OCT corresponded with lymphocytic infiltration on histopathology. On the other hand, conjunctival amyloidosis appeared as heterogeneous, subepithelial lesions with irregular borders that contained hyperreflective opacities on HR-OCT. The hyperreflective opacities seen in the amyloid lesions on HR-OCT likely correspond with the hyperreflective nature of the deposited amyloid material surrounded by less reflective subepithelial tissue.

HR-OCT images of BRLH showed variable infiltration of the subepithelial tissue, at times with homogenous lesions containing dot-like infiltrates like lymphoma and other times with more hyperreflective subepithelial tissue. These morphological differences on HR-OCT of BRLH corresponded with variable degrees of cellular infiltration on histopathology. In cases of BRLH that looked similar to conjunctival lymphoma on HR-OCT, histopathology showed a hypercellular infiltration in the subepithelial tissue. In cases where the BRLH lesions were much more hyperreflective, a paucicellular infiltrate was seen on histopathology. The lymphoproliferative lesions comprise a spectrum of disorders and differences in the configuration of lymphocytes on histopathology for the two entities as well as differences in thickness and surface topographies of the two types of lesions likely explain the variable presentations of BRLH on HR-OCT and histopathology. In addition, a significant difference was found between the mean epithelial layer thickness between BRLH and lymphoma specimens as assessed by in vivo OCT measurements. This finding was only determined with a limited number of BRLH specimens (*n* = 6). Epithelial measurements were not measured histologically due to known variable shrinkage occurring with fixation. While the meaning of this difference is uncertain, it warrants further study with a larger number of cases and histological comparison.

Extensive posterior shadowing was one of the difficulties in interpreting HR-OCT images, especially in the thicker lesions, such as lymphoma. This made determination of the exact depth of the lesion a challenge. This is a limitation of spectral domain OCT imaging – the inability to gauge invasion and depth in thicker lesions. In cases where depth of the lesion was a concern, ultrasound biomicroscopy was subsequently obtained to ensure there was no scleral extension. In addition, in all cases, high quality images were harder to obtain when the lesions were located in the upper or lower fornices as opposed to the bulbar conjunctiva. This occurred more frequently in the cases of amyloidosis.

Our findings expand the number of ocular surface conditions that can be uniquely identified using HR-OCT. [6] For example, we have described that OSSN and pterygia have unique manifestations on HR-OCT that also match corresponding histopathologic findings. HR-OCT can also be used to identify epithelial and stromal degenerations [9], the extent and etiology of peripheral corneal thinning [10], and characteristics of pigmented lesions [11]. OSSN also has distinctive features on HR-OCT, and at our institution, we often reserve conjunctival biopsy for OSSN cases where HR-OCT findings are inconclusive or in cases that are refractory to topical chemotherapeutic treatment.

We found that conjunctival amyloidosis could reliably be distinguished from a lymphoproliferative process on HR-OCT. Conjunctival lymphoma and conjunctival amyloidosis both had distinctive HR-OCT findings; however, the morphology of BRLH on HR-OCT was variable and dependent on the degree of cellular infiltration seen on histopathology. Ultimately, while HR-OCT could capture nuances in lesion morphology, similar to traditional histopathology, cytology and gene re-arrangement were ultimately required to determine if the lymphoproliferative lesions were benign or malignant.

## Conlusions

To conclude, we found that HR-OCT was a helpful adjunctive non-invasive diagnostic modality that can be used to guide the diagnosis of sub-epithelial conjunctival disorders. In the future, larger studies with longitudinal follow-up can further validate this technology’s use with more subtle and challenging lesions.

## Data Availability

Not applicable
